# Inner Amino Acid Contacts Are Key Factors of Multistage Structural Rearrangements of DNA and Affect Substrate Specificity of Apurinic/Apyrimidinic Endonuclease APE1

**DOI:** 10.3390/ijms241411474

**Published:** 2023-07-14

**Authors:** Anatoly A. Bulygin, Victoria N. Syryamina, Aleksandra A. Kuznetsova, Darya S. Novopashina, Sergei A. Dzuba, Nikita A. Kuznetsov

**Affiliations:** 1 Institute of Chemical Biology and Fundamental Medicine, Siberian Branch of Russian Academy of Sciences (SB RAS), Novosibirsk 630090, Russia; skytolya@ya.ru (A.A.B.); sandra-k@niboch.nsc.ru (A.A.K.); danov@niboch.nsc.ru (D.S.N.); 2 Institute of Chemical Kinetics and Combustion, Siberian Branch of Russian Academy of Sciences (SB RAS), Novosibirsk 630090, Russia; v.syryamina@gmail.com (V.N.S.); dzuba@kinetics.nsc.ru (S.A.D.); 3 Department of Natural Sciences, Novosibirsk State University, Novosibirsk 630090, Russia

**Keywords:** base excision repair, nucleotide incision repair, apurinic/apyrimidinic endonuclease, conformational dynamics, active-site plasticity, damaged nucleotide, nucleotide eversion

## Abstract

Apurinic/apyrimidinic endonuclease 1 (APE1) is one of the most important enzymes in base excision repair. Studies on this enzyme have been conducted for a long time, but some aspects of its activity remain poorly understood. One such question concerns the mechanism of damaged-nucleotide recognition by the enzyme, and the answer could shed light on substrate specificity control in all enzymes of this class. In the present study, by pulsed electron–electron double resonance (DEER, also known as PELDOR) spectroscopy and pre–steady-state kinetic analysis along with wild-type (WT) APE1 from *Danio rerio* (zAPE1) or three mutants (carrying substitution N253G, A254G, or E260A), we aimed to elucidate the molecular events in the process of damage recognition. The data revealed that the zAPE1 mutant E260A has much higher activity toward DNA substrates containing 5,6-dihydro-2′-deoxyuridine (DHU), 2′-deoxyuridine (dU), alpha-2′-deoxyadenosine (αA), or 1,*N*6-ethenoadenosine (εA). Examination of conformational changes in DNA clearly revealed multistep DNA rearrangements during the formation of the catalytic complex. These structural rearrangements of DNA are directly associated with the capacity of damaged DNA for enzyme-induced bending and unwinding, which are required for eversion of the damaged nucleotide from the DNA duplex and for its placement into the active site of the enzyme. Taken together, the results experimentally prove the factors that control substrate specificity of the AP endonuclease zAPE1.

## 1. Introduction

Different DNA oxidative lesions are constantly caused by reactive oxygen species produced by cellular metabolism or by exogenous factors. Some of the major adducts forming in DNA are 5,6-dihydro-2′-deoxyuridine (DHU), alpha-2′-deoxyadenosine (αA), and 1,*N*6-ethenoadenosine (εA) [1,2]. Such non-bulky lesions are removed by base excision repair (BER), initiated by DNA glycosylases [3]. The BER pathway in general and DNA glycosylases in particular generate genotoxic intermediates such as apurinic/apyrimidinic (AP) sites, which are next processed by AP endonucleases. Existing evidence indicates that some AP endonucleases can perform incision of the aforementioned lesions in a glycosylase-independent manner [4,5], which is termed nucleotide incision repair (NIR). It has also been reported that AP endonucleases can incise such bulky lesions as 3,*N*4-benzetheno-2′-deoxycytidine and 1,*N*6-benzetheno-2′-deoxyadenosine [6]. Moreover, these DNA adducts are not substrates for any known DNA glycosylase, thereby suggesting that this activity of AP endonucleases is not a “backup” function that is needed in the absence of DNA glycosylases. Additionally, APE1 has 3′-5′-exonuclease activity [7,8,9,10,11,12,13], which is needed in the ATR-Chk1-subpathway of DNA damage response for creating long gaps in DNA [14,15,16,17], and endoribonuclease activity [18,19,20,21,22,23] that was shown to play the role in RNA regulation [24,25,26,27,28].

Because the reason for such wide substrate specificity of AP endonucleases is unclear, in the present study we analyzed NIR activity of the AP endonuclease from *Danio rerio* (zAPE1), which belongs to the Xth structural family. AP endonucleases of this family have been found in many organisms from bacteria to humans. Of note, prokaryotic enzyme Xth and human APE1 (hAPE1) share only 27% identity and evolutionarily accumulated changes have led to the absence of NIR activity in Xth [29]. Nonetheless, another study aimed at elucidating the impact of structural differences in homologous APE1s [10,30] has shown that among APE1-like enzymes from humans, *D. rerio*, *Xenopus laevis*, and *Drosophila melanogaster*, only the latter (called Rrp1) has a reduced NIR activity.

In our previous computational studies on DNA–APE1 complexes [31,32], we have also described interactions between different damaged nucleotides (F-site, DHU, αA, and εA) and closest amino acid residues of APE1-like enzymes from humans, zebrafish, *X. laevis*, and *D. melanogaster.* The main conclusion was that in all cases, damaged bases have steric conflicts with residues 229–231 (the amino acid numbering corresponds to the hAPE1 sequence) when placed inside the active-site pocket. These conflicts can be resolved by a shift of the 229–233 loop (called the “damage recognition loop”) away from the DNA base, which is preceded by the loss of Thr233 O^γ1^–Glu236 O^ε2^ and Ala(Ser)214 N–Gly231 O contacts. After the loss of these bonds, the damage recognition loop moves away from the damaged base of the everted nucleotide, thereby enlarging the inner volume of the active-site pocket and removing the prohibition of corrected placement of the damaged base within the active site.

There are several articles in which point mutations in the aforementioned enzyme regions have been investigated. In ref. [29], researchers compared hAPE1 mutants N174Q and G231C with the Xth enzyme and other homologs. It was shown that these substitutions do not affect activity toward a DNA substrate containing an F-site or αA. Two studies have addressed effects of α8-loop substitutions in hAPE1 on activity toward an F-site–containing substrate (F-substrate) [33,34]. The results revealed that there is a set of substitutions (N226A, K227L, K228T, and N229A) that have a negative impact on binding affinity but a positive influence on the catalysis rate, thus resulting in approximately the same overall efficacy as in the WT enzyme.

In the current study, using the zAPE1 enzyme from zebrafish along with pulsed double electron–electron resonance (DEER, also known as PELDOR) spectroscopy and pre–steady-state kinetic analysis, we aimed to gain a deep insight into the molecular events occurring during damage recognition by this enzyme. The functions of three amino acid residues—Asn253, Ala254, and Glu260—of zAPE1 (corresponding to Asn229, Ala230, and Glu236 of hAPE1), which are located in the damage recognition loop, were experimentally estimated in this work and allowed us to verify our previous computational prediction [31,32]. That prediction highlighted their importance in the molecular mechanism of substrate specificity toward NIR substrates. A point mutation (N253G, A254G, or E260A) was introduced into zAPE1, and the mutants were characterized by means of catalytic efficiency toward DNA containing an F-site, DHU, dU, αA, or εA. Real-time conformational changes of damaged FRET-labeled DNA in the process interaction with the tested mutant enzymes revealed a complex mechanism of DNA transformations involving sequential stages of a distance decrease and increase between an emitter and quencher in a Forster resonance energy transfer (FRET) pair. DEER analyses of rigid-linker spin-labeled DNA duplexes in a catalytic complex with WT zAPE1 indicated unwinding of the DNA duplex. Taken together, our results revealed a multistage process of damaged-nucleotide recognition including sequential steps of DNA bending, DNA unwinding, and lesion eversion leading to the widening of the active-site pocket and finally resulting in catalytic-complex formation.

## 2. Results and Discussion

### 2.1. Selection of Amino Acid Residues Facilitating the Widening of the Active-Site Pocket

**Figure 1:**
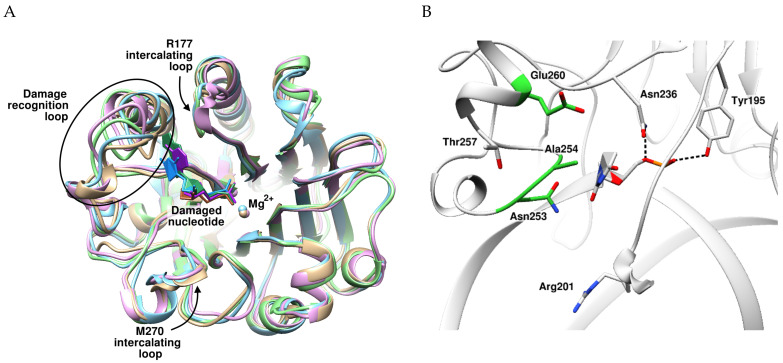
(**A**) Superposition of models of zAPE1 complex with DNA containing an F-site (gold), DHU (blue), εA (green), or αA (magenta). (**B**) Close-up view of amino acid residues responsible for flexibility of the damage recognition loop. Residues chosen for substitution are highlighted in green.

In previous studies [31,32], computational prediction of plasticity of the active-site pocket has revealed that the loop consisting of residues 253 to 257 is required for accommodation of different nucleotides containing damaged bases (Figure 1A). Therefore, the main principle behind the choice of amino acid residues for substitution was to increase the flexibility and mobility of loop 253–257, which in turn should increase the efficiency of NIR activity. To experimentally verify changes in the cleavage of damaged DNA owing to the increased flexibility of the damage recognition loop, it was decided to create mutant zAPE1 enzymes containing substitution N253G, A254G, or E260A (Figure 1B). Two of the chosen amino acids, Asn253 and Ala254, engage in direct interactions with a damaged base, whereas Glu260 may be responsible for the flexibility of the damage recognition loop in the course of damaged-base eversion.

### 2.2. Comparison of F-Site Cleavage Efficiency by Mutant zAPE1 Enzymes

**Figure 2:**
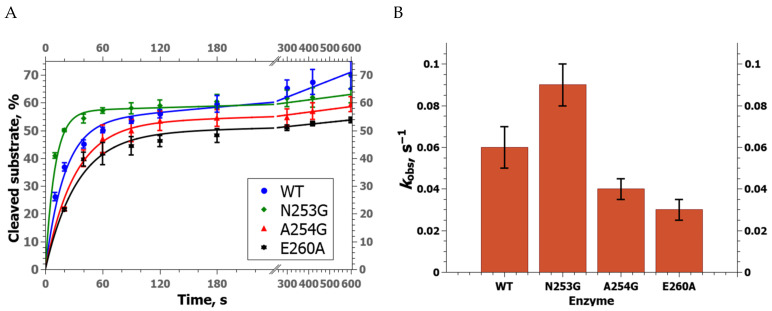
(**A**) Kinetic curves of product accumulation in the course of F-substrate cleavage by the WT zAPE1 enzyme or by its mutants under steady-state conditions at [E] = 0.04 μM and [S] = 1.0 μM. (**B**) A comparison of observed rate constants (mean ± SD, s^−1^) of the AP endonuclease reaction of the zAPE1 enzymes.

A DNA substrate containing an F-site (F-substrate) was used to estimate the rate of product accumulation during interaction with either WT zAPE1 or its mutant under steady-state conditions (Figure 2A). It was found that N253G zAPE1 has the highest observed rate constant of the F-substrate cleavage (Figure 2B) with *k*_obs_ = 0.09 ± 0.01 s^−1^. WT zAPE1 ranked second (*k*_obs_ = 0.06 ± 0.01 s^−1^), while substitutions A254G and E260A unexpectedly led to a notable decrease in the observed rate constant (0.040 ± 0.005 and 0.030 ± 0.005 s^−1^, respectively). The data revealed that under conditions of multiple turnovers of the enzyme, when the dissociation of the enzyme–product complex could be important for product accumulation, the difference in the observed rate constants was only ≤3-fold, indicating that the tested amino acid substitutions do not affect this stage significantly.

To analyze F-substrate cleavage under single-turnover conditions, changes in the FRET fluorescence signal between emitter FAM and quencher BHQ1 located at the ends of the DNA duplex were recorded on a stopped-flow spectrophotometer. According to the literature [35,36], an increase and decrease in the FRET signal with time indicates an increase and decrease in the distance between the FAM fluorophore and the BHQ1 quencher, respectively, as a consequence of the formation of the enzyme–substrate complex, subsequent cleavage of the F-site, and dissociation of the enzyme–product complex.

**Figure 3:**
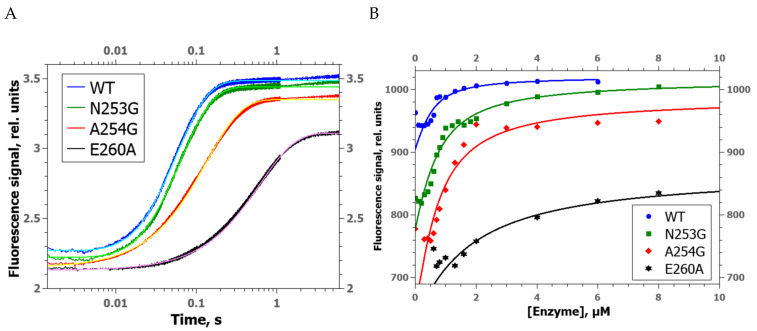
(**A**) Kinetic curves of FRET signal changes in the course of interaction of zAPE1 enzymes with the F-substrate under single-turnover conditions at [E] = 2.0 µM and [S] = 1.0 µM. (**B**) MST titration of F-site–containing DNA by zAPE1 enzymes.

All obtained kinetic curves of FRET signal changes had similar shapes having a barely noticeable initial decrease phase, (reflecting complex formation) and a high-amplitude phase of signal growth (corresponding to the accumulation of the product; Figure 3A). These curves were fitted to an exponential equation (Equation (7)) to estimate observed rate constants of both phases (Table 1). Because the changes in the FRET signal during the formation of the enzyme–substrate complex have a small amplitude, even a slight distortion of DNA-binding efficiency by amino acid substitutions hampers the calculation of parameters of this process owing to the loss of this phase in the kinetic curves. Indeed, it was revealed that the initial decrease in the FRET signal was insufficient for correct calculation of the observed rate constant reflecting enzyme–DNA complex formation in the case of substitutions A254G and E260A. Nevertheless, the data showed that mutants A254G and E260A, but not N253G, are catalytically slower than the WT enzyme under single-turnover conditions. Similarity of the catalytic activity between the N253G mutant and WT enzyme suggested that the slightly higher activity of N253G under steady-state conditions is most likely explained by some destabilization of the enzyme–product complex by the replacement of the Asn253 residue.

To determine the contribution of the DNA-binding ability of the mutant enzymes to the alteration of the F-site cleavage efficiency, association constants of the enzyme–substrate complexes were determined by microscopic thermophoresis (MST) (Figure 3B). The association constants for WT, N253G, and A254G were found to be approximately equal (Table 1), whereas E260A manifested a ~4-fold lower value, implying distortion of DNA binding by this substitution. It should be noted that this decrease in the association constant matches the pattern of the observed rate constant *k*_2_, which characterizes the DNA incision process. Therefore, the decrease in the DNA-binding ability of the zAPE1 E260A mutant is one of the factors reducing the catalytic activity.

**Figure 4:**
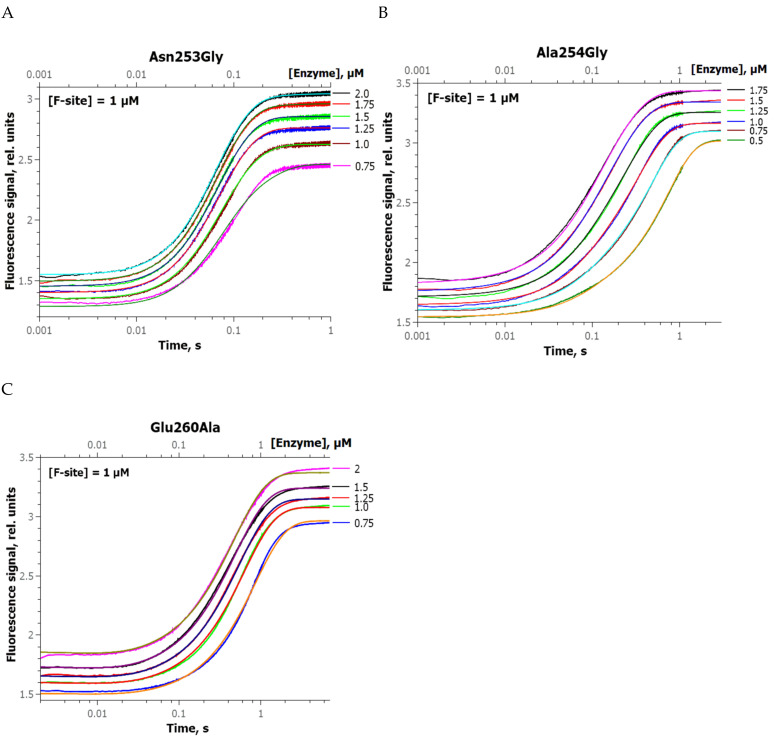
Changes in the FRET signal during the interaction of zAPE1 N253G (**A**), A254G (**B**), or E260A (**C**) enzyme with the F-substrate. [S] = 1.0 µM, enzyme concentrations are shown in the panels. Theoretical curves (smooth traces) were obtained by the global fitting procedure in the DynaFit 4 software.

To further characterize the role of the selected amino acid residues at individual stages (F-site recognition, formation of a catalytically active complex, catalysis, and enzyme–product complex dissociation), conformational alterations of the F-substrate during interaction with the zAPE1 enzymes were recorded at various concentrations of the reactants. This approach allowed us to determine individual rate constants in the kinetic mechanism. A set of kinetic curves was obtained by the stopped-flow technique, reflecting a change in the FRET signal in the course of interaction of mutant zAPE1 enzymes with the F-substrate (Figure 4).

Kinetic Scheme 1, which describes our experimental data, includes the reversible stage of enzyme–substrate binding, the irreversible catalytic stage, and the reversible stage of dissociation of the enzyme–product complex. Theoretical fitting traces were obtained in the DynaFit 4 software [37], which made it possible to calculate the corresponding rate constants contained in this kinetic scheme (Table 2). During the construction of the theoretical curves, *K*_a_, which corresponds to the enzyme–substrate complex formation, was set on the basis of the MST results. It was found that the catalytic constant *k_cat_* decreases from 13 to 4 s^−1^ in accordance with the same pattern as that of the observed rate constant *k*_2_ (Table 1). For dissociation constants of the complex with the product, *K_p_*, as expected, the pattern was opposite to that of the substrate-binding constant.

Scheme 1 F-substrate processing by zAPE1 enzymes.

$$E+S\;\begin{array}{c} k_{1}\\ ⇄\\ k_{-1} \end{array}ES\;\overset{k_{cat}}{\to }\;EP\;\begin{array}{c} K_{p}\\ ⇄ \end{array}\;\;E+P$$

where *E* is the enzyme, *S* is the DNA substrate, *ES* is the enzyme–substrate complex, *EP* is the enzyme–product complex, and *P* is the reaction product.

A comparison of the data obtained under single-turnover conditions suggested that both A254G and E260A mutants have lower catalytic constants *k_cat_*. This effect of substitutions together with the decrease in the DNA-binding ability (most pronounced in the case of zAPE1 E260A) led to a decrease in the total cleavage efficiency of the mutant enzymes toward the F-substrate, except for N253G, which was very close to the WT in this regard. Moreover, the N253G substitution somewhat facilitated the enzyme dissociation from the product as compared with the WT enzyme. The A254G substitution had a slight inhibitory effect on all stages of interaction with the F-substrate.

### 2.3. Efficiency of Cleavage of Damaged-Base-Containing DNA Substrates

**Figure 5:**
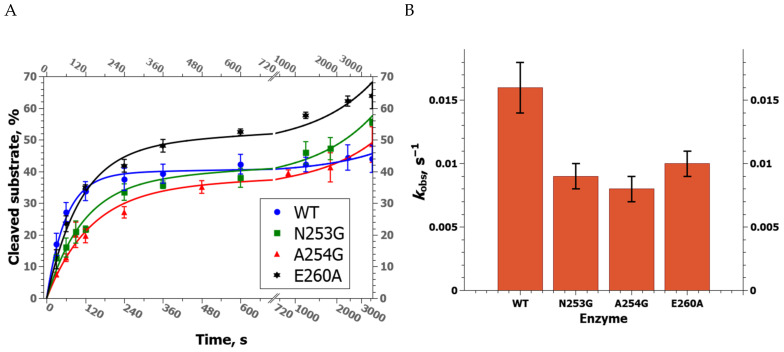
(**A**) Product accumulation kinetic curves of DHU-substrate cleavage by WT zAPE1 and its mutants under steady-state conditions at [E] = 0.2 μM and [S] = 1.0 μM. (**B**) A comparison of observed rate constants (mean ± SD, s^−1^) of the AP endonuclease reaction of zAPE1 enzymes.

**Figure 6:**
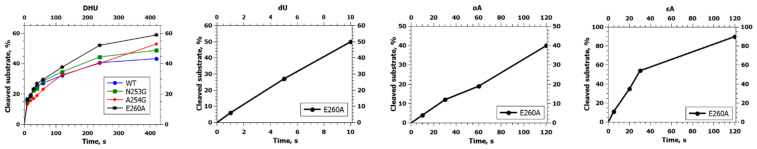
Kinetic curves of product accumulation in the course of cleavage of NIR substrates by zAPE1 E260A under steady-state conditions. [E] = 2.0 μM, [S] = 1.0 μM.

**Figure 7:**
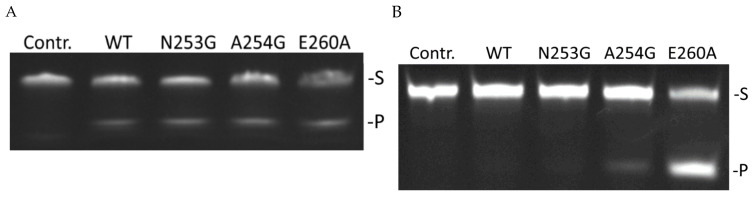

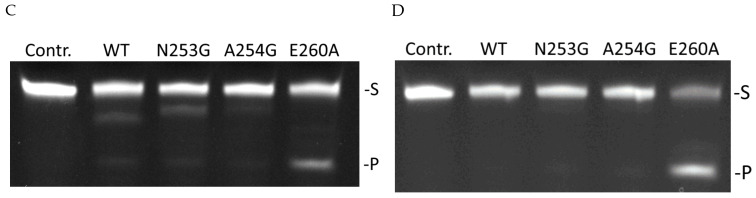
Polyacrylamide gel electrophoresis (PAGE) analysis of product accumulation under the action of zAPE1 enzymes after 40 s for the DHU-substrate (**A**), 10 min for the dU-substrate (**B**), 60 min for the αA-substrate (**C**), and 30 min for the εA-substrate (**D**). [E] = 2.0 μM, [S] = 1.0 μM. Lane “Contr.” denotes DNA substrate mobility without the enzyme treatment.

DHU-containing DNA (hereafter: “DHU-substrate”), as one of the most efficient NIR substrates for APE1-like endonucleases [30,35,38], was used to determine the kinetics of product accumulation during interaction with tested mutants of zAPE1 (Figure 5A). The data showed that all mutant enzymes are 1.5–2.0-fold slower than the WT enzyme (Figure 5B). It is noteworthy that in the case of zAPE1 E260A, which has the worst efficiency toward the F-substrate, the rate of accumulation of DHU-substrate cleavage products was the same as that of the WT enzyme. Moreover, it was demonstrated that total cleavage efficiency during 60 min of the reaction was the highest for this mutant of the enzyme. Therefore, zAPE1 E260A, as the most active toward the DHU-substrate, was used to find the optimal time of the reaction with other NIR substrates, containing dU, αA, or εA, under single-turnover conditions (Figure 6). The results enabled us to select a set of time points for direct comparison of all mutant enzymes in terms of total enzyme efficiency toward DHU-, dU-, αA-, or εA-containing DNA with an excess of the enzyme over DNA (Figure 7).

**Figure 8:**
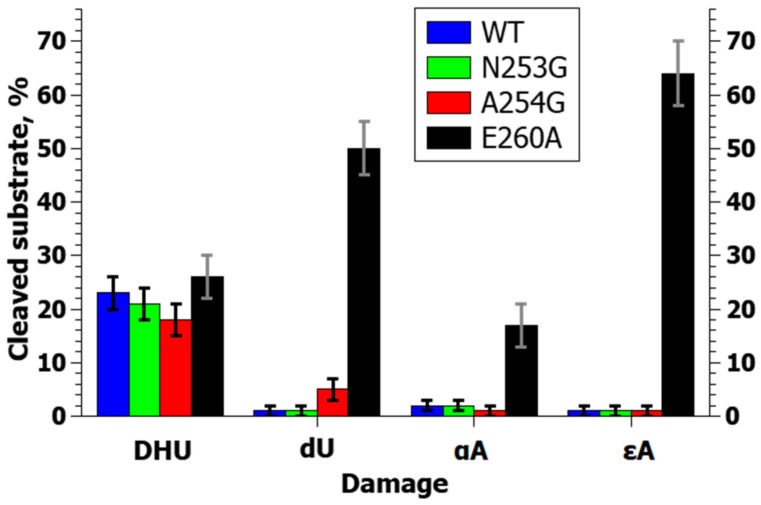
Efficiency of NIR substrate cleavage by zAPE1 mutants under selected conditions. In this case, the whiskers define the interval in which the results of different repetitions of the experiment lie.

A comparison of enzyme activity toward NIR substrates clearly indicated that zAPE1 E260A is capable of much more efficiently cleaving any of the tested NIR substrates in comparison with the WT enzyme and the other tested enzymes, except for the DHU-substrate, where the efficiency was similar (Figure 8). On the other hand, the mutants N253G and A254G showed enzymatic activity toward NIR substrates that was similar to that of the WT. The efficiency of NIR activity of these enzymes decreases in the order DHU >> αA ≈ εA ≈ dU; however, the A254G mutant was slightly more active toward the dU-substrate.

At the same time, zAPE1 E260A was the only mutant that was capable of incising the εA-substrate with a considerable yield: the proportion of the εA-substrate cleaved by zAPE1 E260A was ~64%. The alpha anomer of adenosine, which was barely cleaved (~2%) during the 60 min reaction by enzymes WT and N253G, also was noticeably better cleaved by the E260A mutant (~17%). In addition, the mutant E260A better cleaved the dU-substrate, which was difficult for WT, with the highest degree of cleavage of ~50%. The efficiency of NIR activity of the mutant E260A decreases in a different order (DHU ≈ dU > εA > αA) and was much higher than that of the other tested enzymes.

### 2.4. Stopped-Flow Fluorescence Measurement

**Figure 9:**
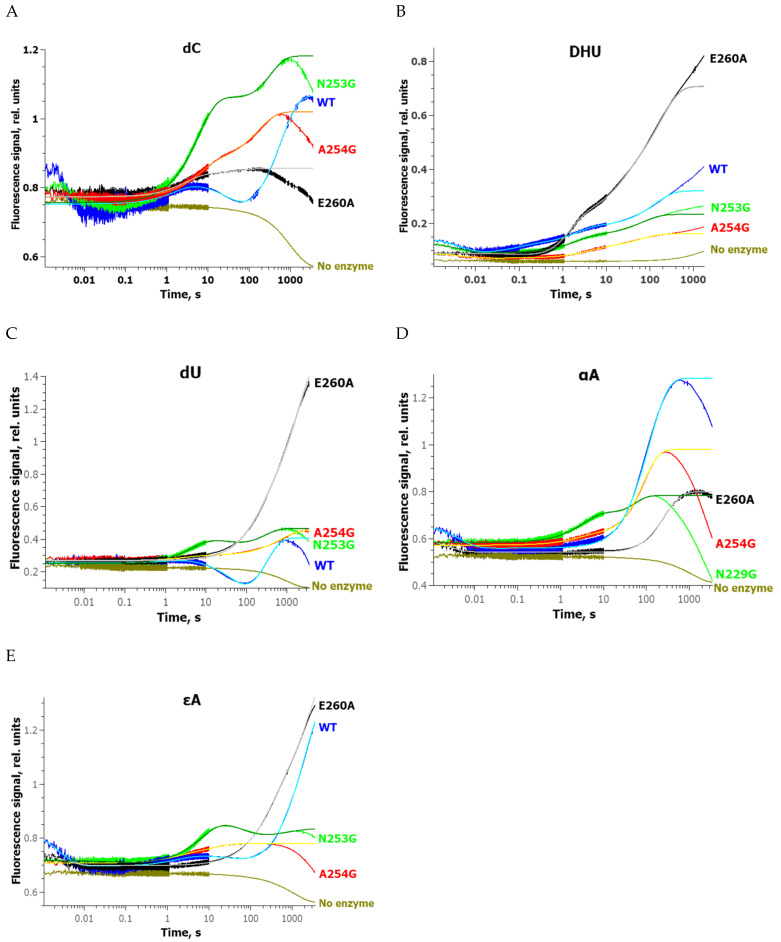
Changes of the FRET signal during the interaction of zAPE1 enzymes with undamaged DNA (**A**) or damaged DNA containing DHU (**B**), dU (**C**), αA (**D**), or εA (**E**). [E] = 2.0 μM, [S] = 1.0 μM.

Analyses of conformational changes in undamaged DNA and NIR substrates were performed on all the investigated enzymes under single-turnover conditions (Figure 9). The obtained kinetic curves revealed that the enzyme’s interaction with undamaged DNA or NIR substrates leads to sequential multistep changes of the FRET signal. These findings indicated a complex kinetic mechanism of nonspecific binding to undamaged DNA and the formation of catalytic complexes with cleavable NIR substrates.

Kinetic curves reflecting changes in the signal in experiments with undamaged DNA contained two growth phases, while the curves corresponding to the WT and to the Asn229Gly mutant contained an additional intermediate decline phase (Figure 9A). In the case of the DHU-substrate, which proved to be cleavable by all the tested zAPE1 enzymes, all curves had one phase of a signal drop-off in the initial part of the graph and two pronounced phases of growth in the rest of the graph (Figure 9B). It is possible that the substantial increase in the FRET signal after 10 s reflects the catalytic stage of the reaction, which leads to the formation of the product and the subsequent process of dissociation of the enzyme–product complex; this rise of the signal can be attributed to the increase in the distance between members of the FRET pair of dyes. The kinetic curves characterizing the interaction of enzymes with the dU-substrate had shapes similar to those obtained for undamaged DNA (Figure 9C). Of note, in the case of the E260A mutant, which is one of the most active toward this substrate, the last phase of growth had a much greater amplitude as compared to the other enzymes. Nonetheless, analysis of the kinetic curves obtained for αA- (Figure 9D) and εA-substrates (Figure 9E), which both are cleaved slowly by any tested enzymes, revealed a discrepancy between the growth of the FRET signal and the efficiency of DNA substrates’ cleavage. Indeed, a comparison of the curves for the αA-substrate indicated that the highest amplitude of FRET signal growth was registered for the WT, which has lower efficiency of DNA cleavage in comparison with E260A during 60 min of the reaction (Figure 8). The same conclusion can be drawn about the εA-substrate, which was best cleaved by the Asn260Gly mutant during 30 min of the reaction. It is possible that FRET detection for slowly cleaved substrates does not reflect the enzymatic process owing to limitations of the technique, such as bleaching of fluorophores, detectable in all control kinetic curves.

Nevertheless, it could be concluded that the analysis of conformational changes in DNA containing damaged bases clearly outlined the multistep DNA rearrangements during the formation of an enzyme–substrate complex and its transformation into the catalytically active state. It can be theorized that the differences in conformational rearrangements of DNA, and in their cleavage efficiency, can be attributed to sequential processes in the course of the catalytic-complex formation, which requires the eversion of the damaged nucleotide from the DNA duplex and placement of the lesion into the active site of the enzyme.

### 2.5. EPR Measurements

Continuous wave (CW) electron paramagnetic resonance (EPR) spectra of the DNA duplexes with various lesions and zAPE1 as recorded at 100 K were typical for an immobilized nitroxide label [39] and are given in the Supplementary Materials (Figure S1). Among all the samples, there were no discernable differences. Furthermore, these spectra did not contain a pronounced broadening of individual linewidths, which means that the distances between spin labels exceed 1.8–2.0 nm [40,41], thus making CW EPR insensitive to such structural changes.

The weak dipole–dipole coupling was probed by pulsed dipolar EPR spectroscopy, namely, DEER (also called PELDOR) [42]. The raw DEER time traces obtained at different frequency offsets (see Experimental) are given in the Supplementary Materials, Figures S2–S4. Note that these data did not reveal any visible dependence on the frequency offset (see Figures S2–S4, panels A and B), thereby pointing to the absence of orientation selectivity effects [43,44] in our case.

**Figure 10:**
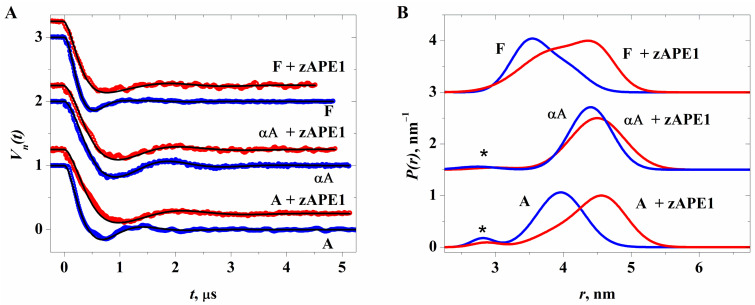
(**A**) Normalized three-pulse DEER time traces (circles, noisy lines) and their best fits (smooth lines) obtained by multi-Gaussian Monte Carlo simulations. The curves are shifted upwards for clarity. (**B**) The distance distribution functions *P*(*r*) derived for DNA duplexes without zAPE1 (blue curves) and with zAPE1 (red curves). The asterisks mark artefact peaks near 2.8 nm (see the main text).

The DEER data, processed and analyzed as described in the DEER data processing subsection below, are given in Figure 10A. Their cosine-Fourier transforms are presented in Figures S2–S4. The simulated DEER time traces with the *P*(*r*) functions derived for each compound are represented by black curves in Figure 10A.

Figure 10B depicts the obtained distance distribution functions *P*(*r*), with the parameters given in Table 1; data on the duplexes without zAPE1 were taken from [45]. For the undamaged DNA and the αA-substrate without and with the zAPE1 protein, one can see a tiny peak at 2.6–2.8 nm, representing a fraction less than 4%. This peak can be readily ascribed to a residual ^2^H modulation of the echo signal because of a partial overlap between detection and pumping pulses. This artefact was ignored in the further analysis.

One can see in Figure 10B and in Table 3 that for the undamaged duplex at the DNA:zAPE1 ratio of 1.25:1.00, the distance distribution function *P*(*r*) shows bimodality, with the major peak at 4.60 nm and the smaller peak at 3.94 nm. The latter may be ascribed to the DNA duplex without protein [45]. In this case, the major peak implies elongation of the mean distance between spin labels in the presence of zAPE1. Note also, the broadening of this peak in the case of duplexes containing a lesion: Figure 10B, Table 3. For the F-substrate, the bimodality with almost the same fractions was also registered in the complex with zAPE1.

Note that the obtained DEER data are in good agreement with the previous results on rigid spin labels connected to an inner-chain nucleobase [45], which also showed the elongation in the complex with the bacterial AP endonuclease Nfo. This elongation in ref. [45] was interpreted as DNA unwinding. Here, we can also conclude that in the zAPE1 complex with damaged DNA, the unwinding of the double helix occurs too.

Therefore, the obtained kinetic FRET data, which reflect changes of the distance between the emitter and quencher, could be explained not only by the DNA-bending process but also includes DNA unwinding if we take into account the very complicated and unpredictable behavior of FRET signal changes.

## 3. Materials and Methods

### 3.1. Site-Directed Mutagenesis and Protein Purification

Plasmid pET28c-zAPE1 [10,30] containing the *zAPE1* gene carrying an N-terminal His_6_ tag was used to construct mutants of the enzyme containing substitution N253G, A254G, or E260A. For expression of the recombinant proteins, 0.5 L of culture of *E. coli* ArcticExpress (DE3) cells (Invitrogen, Waltham, MA, USA) (in 2xYT broth) carrying the pET28c-zAPE1 construct was grown at 50 μg/mL kanamycin, 50 μg/mL gentamicin, 10 μg/mL tetracycline and 37 °C until absorbance at 600 nm (A_600_) reached 0.6–0.7. APE1 expression was induced overnight with 0.3 mM isopropyl-β-D-thiogalactopyranoside at 18 °C. The cells were then harvested by centrifugation and resuspended in a buffer (20 mM HEPES-KOH pH 7.6, 40 mM NaCl) followed by cell lysis using a French press. The homogenate was centrifuged at 40,000× *g* for 40 min, and the supernatant was passed through a column packed with 50 mL of Q-Sepharose Fast Flow resin (Cytiva, GE Healthcare Life Sciences, Marlborough, MA, USA) pre-equilibrated in the same buffer. All purification procedures were carried out at 4 °C. The flow-through fractions containing zAPE1 were pooled and loaded onto a 1 mL HiTrap™ Chelating HP column (Cytiva, GE Healthcare Life Sciences, Marlborough, MA, USA). Bound proteins were eluted with a 20–600 mM imidazole gradient. The protein concentration was measured by the Bradford method; stock solutions were stored at −20 °C with 50% glycerol.

### 3.2. DNA Substrates

The sequences of the DNA substrates employed in this work are presented in Table 4. Oligodeoxynucleotides (ODNs) were synthesized by standard phosphoramidite methods on an ASM-800 synthesizer (BIOSSET Ltd., Novosibirsk, Russia) using phosphoramidites purchased from Glen Research or ChemGenes. The synthetic ODNs were purified by HPLC on an Waters 600E chromatograph (USA) and a Phenomenex Prodigy column (ODS-3, 5u, 100 Å, 5 μm), 4.6 × 250 mm, via a linear gradient of acetonitrile (0 → 30%) in the presence of 20 mM triethylammonium acetate (pH 7.0) for 60 min at a flow rate of 2 mL/min. Fractions containing ODNs were dried in vacuum, dissolved in water, and subjected to precipitation with 2% LiClO_4_ in acetone. After washing with pure acetone and drying, the ODN precipitates were dissolved in water and stored at −20 °C until experiments. Concentrations of the ODNs were determined by means of A_260_. All DNA duplexes were prepared by annealing of modified and complementary strands at the 10:10 μM ratio in reaction buffer. The latter (50 mM Tris-HCl pH 7.5, 50 mM KCl, 1.0 mM EDTA, 1.0 mM DTT, and 7% of glycerol) was supplemented with 5.0 mM MgCl_2_ for F-substrates and with 1.0 mM MgCl_2_ for DHU-, U-, αA-, and εA-substrates.

### 3.3. DEER Sample Preparation

The DNA duplexes for DEER measurements were obtained by mixing single-stranded ODNs (undamaged or containing a lesion) with a complementary TEMPO-labeled ODN at room temperature in binding buffer consisting of 10 mM Na_2_HPO_4_ (pH 7.5) and 140 mM NaCl. The DNA duplexes were dissolved in a water–glycerol 1:1 (*v*/*v*) mixture (^2^H_2_O and C_3_^2^H_8_O_3_). The concentrations of spin-labeled DNAs were 2.0 × 10^−4^ M. Samples containing the zAPE1 protein were prepared by dilution of an appropriate concentration of the DEER-X-substrate with a deuterated water/deuteroglycerol solution of the protein obtained by a buffer exchange procedure using an Amicon ultra 3 K column (Merck). The final concentrations of spin-labeled DNA and zAPE1 were 1.0 × 10^−4^ M for F-site and αA lesions, and the DNA/zAPE1 ratio was 1.25 × 10^−4^/1.0 × 10^−4^ for the undamaged DNA duplex. The samples were placed in EPR tubes and quickly frozen in liquid nitrogen. All samples upon freezing formed a transparent glass. The outer diameter of the EPR tubes was 2.9 mm, and each sample’s length did not exceed 4 mm. Between the measurements, the samples were stored in liquid nitrogen.

### 3.4. EPR Measurements

CW X-band EPR spectra were recorded using a Bruker ELEXSYS E580 spectrometer equipped with a Bruker ER 4118 X-MD-5 resonator. Microwave (MW) power was attenuated to −35 dB, with the calibrated total 200 mW output MW power of a Gunn diode. Modulation frequency and modulation amplitude were set to 100 kHz and 0.05 mT, respectively. The sweep width was 15 mT. The time constant was 20.48 ms, and conversion time was 40.96 ms.

Pulse EPR experiments were conducted with a Bruker ER 4118 X-MS-3 resonator. Three-pulse DEER measurements [42,46,47] were performed by means of the pulse sequence *t_pulse_ − t − t_pump_ −* (*τ − t*) *− 2t_pulse_ − τ − detection*, where *t_pulse_* and *t_pump_* are durations of the echo-forming pulses acting at the MW frequency *ν_A_* and of the pumping pulse acting at the MW frequency *ν_B_*, with *t_pulse_* = 16 ns and *t_pump_* = 28 ns. The MW power was adjusted to obtain a maximal echo signal. Delay *t* for the pumping pulse was initially set to 240 ns before the first pulse and then scanned by 12 or 24 ns steps. The frequency offset was chosen symmetrically with respect to the resonator dip, and pumping frequency *ν_B_* was chosen to correspond to the maximum of the echo-detected EPR spectrum. The offset *ν_A_* − *ν_B_* was varied from 90 to 40 MHz to explore the effects of so-called orientational selectivity [43,44]. The MW power of the pumping pulse was adjusted by nutation measurements at *ν_A_ = ν_B_* to maximize the inverse echo signal. Delay *τ* was set to 6.5 or 5.8 μs. The signal distortion upon the passage of the pumping pulse through the first detecting pulse was eliminated by means of the measuring scheme described in refs. [47,48], and the initial delay (with the setting *t* = 0) was determined as described in [49].

Temperature was controlled in both CW and pulse EPR measurements with an ER 4118 CF cryostat (Oxford Instruments). The CW EPR measurements were performed at 100 K using a cold nitrogen stream. The pulse EPR measurements were taken at 70 K with the help of liquid nitrogen and the cryostat pumping.

### 3.5. DEER Data Processing

In DEER spectroscopy of double-spin-labeled molecules, experimental signal time trace *V*(*t*) is compared with the theoretical expression:

(1) $$\begin{array}{l} V(t)=V(0)V_{inter}(t)V_{intra}(t),\\ V_{inter}(t)=\exp(-\alpha t^{\xi })\\ V_{intra}(t)=(1-\lambda <\cos\left(\frac{D}{r^{3}}(1-3\cos^{2}\theta )t\right)>) \end{array}$$

where *V_inter_*(*t*) describes intermolecular decay between different biradicals (so-called background decay), with constant *α* proportional to the biradical concentration, and exponent *ξ,* in our case, is employed to take into account the effect of excluded volume [41,46,50]; *V_intra_*(*t*) describes the intramolecular decay, in which *λ* determines the probability of excitation by the pumping pulse, *r* is the spin–spin distance in the biradical, *θ* is the angle between vector **r** and the magnetic field, and the angular brackets mean space averaging. Factor *D* in Equation (1) is expressed as

(2) $$D=\frac{\mu_{0}g_{A}g_{B}\mu_{B}^{2}}{4\pi \hbar }$$

where *g_A_* and *g_B_* are *g*-values of the two spins, *μ_B_* is the Bohr magneton, and *ħ* is the reduced Planck constant.

The space averaging in Equation (1) is performed over angles *θ* and pair distance distribution function *P*(*r*):

(3) $$<\cos\left(\frac{D}{r^{3}}(1-3\cos^{2}\theta )t\right)>=\int_{0}^{\pi /2}\sin\theta d\theta \int_{0}^{\infty }P(r)dr\cos\left(\frac{D}{r^{3}}(1-3\cos^{2}\theta )t\right)$$

where function *P*(*r*) is assumed here to be normalized, so that $\int_{0}^{\infty }P(r)dr=1$.

The main task of DEER spectroscopy is to obtain the *P*(*r*) function by solving this integral equation. To this end, it is necessary to extract function *V_intra_*(*t*) in Equation (1) from the experimental time dependence of DEER signal *V*(*t*). This can be carried out by finding function *V_inter_*(*t*). Exponent *ξ* in this function was adjusted as follows. Its value was varied, the corresponding *V_inter_*(*t*) function was subtracted from the *V*(*t*) function, and the resulting *V_intra_*(*t*) function was normalized as described below and subjected to the Fourier cosine transform. In the frequency domain, a Pake-like pattern is observed that is typical for two magnetic dipoles coupled by a dipole–dipole interaction. Then, the *ξ* value was adjusted by minimization of the artefact at zero frequency. The best-fit values were found to lie between 1.0 and 1.05. After that, normalized experimental dependence *V_n_*(*t*) was obtained [51] as

(4) $$V_{n}(t)=\frac{V_{intra}(t)-V_{intra}(\infty )}{V_{intra}(0)-V_{intra}(\infty )}$$

and *V_n_*(*t*) was taken as the left side of theoretical Equation (3).

For solving the resulting integral equation, different approaches can be used [52,53,54,55,56]. Here, the multi-Gaussian Monte Carlo algorithm was employed [55], which offers fast convergence in the frequency domain. Note that this approach has been shown [38,45,55] to yield results very similar to those obtained with widely used DeerAnalysis2006 software [53]. The spin–spin distance distribution function within the multi-Gaussian approach [55] was assumed to be a composition of three Gaussians:

(5) $$P\left(r\right)=\sum_{i=1}^{3}A_{i}\frac{1}{\sqrt{2\pi \delta r_{i}^{2}}}\exp\left(-\frac{{\left(r-<r_{i}>\right)}^{2}}{2\delta r_{i}^{2}}\right)$$

where the set of parameters *A_i_*, *r_i_*, and *δ_i_* (*i* = 1, 2, or 3) was independently varied upon fitting (taking into account that *A*_1_ + *A*_2_ + *A*_3_ = 1). For all simulated traces, the fraction of one out of three Gaussians was found to be less than 4%.

The dispersions (*δr*) (see Table 3) were estimated by scanning the χ^2^ surface [52], with estimation of a signal-to-noise ratio for the *V_n_*(*t*) function as 50–100.

### 3.6. PAGE Analysis of the DNA Cleavage

Product accumulation in the course of interaction of an enzyme and a DNA substrate was registered by PAGE. To start a reaction between the enzyme and substrate, their solutions were mixed in a suitable buffer at 25 °C for BER-substrates and at 37 °C for NIR-substrates. BER buffer consisted of 50 mM Tris-HCl (pH 7.5), 50 mM KCl, 5 mM MgCl_2_, 1 mM dithiothreitol, 1 mM EDTA, and 7% glycerol (*v*/*v*). NIR buffer was composed of 50 mM Tris-HCl (pH 7.5), 50 mM KCl, 1 mM MgCl_2_, 1 mM dithiothreitol, 1 mM EDTA, and 7% glycerol (*v*/*v*). At a certain time point, the reaction was immediately quenched with a gel-loading dye containing 8 M urea and 25 mM EDTA and was loaded onto a 20% polyacrylamide/8 M urea gel. The gels were visualized using an E-Box CX.5 TS gel-documenting system (Vilber Lourman, Collégien, France) and quantified in the Gel-Pro Analyzer 4.0 software (Media Cybernetics, Rockville, MD, USA).

Based on the results of PAGE analysis, kinetics of accumulation of the reaction product depending on time were obtained. Kinetic curves were approximated as exponential Equation (6):

[Product] = *A* × (1 − exp(−*k*_obs_ × *t*)) + *k*_lin_ × *t* (6)

where *A* is amplitude (%), *k*_obs_ is the observed rate constant (s^−1^), and *k*_lin_ is the rate of linear accumulation (%/s).

### 3.7. Stopped-Flow Fluorescence Detection of Interactions with DNA Substrates

Stopped-flow measurements with fluorescence detection were carried out by means of SX.18MV and SX.20 stopped-flow spectrometers (Applied Photophysics Ltd., Leatherhead, UK) as described in [22,57,58,59]. To detect the fluorescence of 6-carboxyfluorescein (FAM) present in the ODN, wavelength *λ*_ex_ = 494 nm was utilized to excite this residue, and its emission was analyzed at *λ*_em_ > 530 nm (Schott filter OG-530, Mainz, Germany). The concentration of the FRET-F-substrate in the experiments with FAM fluorescence detection was 1.0 μM, and the enzyme concentration varied from 0.25 to 2.0 μM. Typically, each trace shown is the average of three independent experiments. All the experiments with the F-substrate were conducted at 25 °C in BER buffer and with NIR substrates at 37 °C in NIR buffer.

### 3.8. Global Fitting of the Stopped-Flow Data

The individual kinetic curves were fitted to functions in the form of exponential polynomials:

(7) $$F\left(t\right)=\underset{n}{\sum }A_{n}e^{-k_{n}t}$$

where *A_n_* are the amplitudes of the *n*th phase (relative units), and *k_n_* are the observed rate constants of the *n*th phase (s^−1^).

The sets of kinetic curves obtained at different concentrations of the reactants were analyzed in the DynaFit 4 software (BioKin, Pullman, WA, USA) [37]. Concentrations of each species in the mechanisms are described by a set of differential equations according to a kinetic scheme. The software performs numerical integration of a system of ordinary differential equations with subsequent nonlinear least-squares regression analysis. In the fits, the values of all relevant rate constants for the forward and reverse reactions of the proposed kinetic scheme were optimized. A kinetic scheme was chosen as optimal when standard deviations of the data residuals after fitting to the proposed scheme were minimal.

### 3.9. MST Titration

The association constants of zAPE1 enzymes with FRET-F/G DNA substrates were determined by the MST method using a Monolith NT.115 system (Nano-Temper Technologies, Munich, Germany). ODN concentrations were 0.5 μM, while the concentrations of the enzymes varied from 0.1 to 10 μM. Reaction mixtures were incubated at 25 °C in a buffer composed of 50 mM Tris-HCl (pH 7.5), 50 mM KCl, and 2.0 mM EDTA.

(8) $$MST_{signal}=F_{backgr}+F_{ampl}\times \frac{\left[zAPE1\right]}{1/K_{a}+\left[zAPE1\right]}$$

where *F_backgr_* is the background signal, *F_ampl_* is *MST_signal_* variation amplitude, and *K_a_* is the equilibrium association constant (*M*) of an enzyme–substrate complex.

### 3.10. Statistical Analysis

All theoretical curves approximating the data from DNA cleavage experiments, mean values, and their errors and standard deviations were obtained in the QtiPlot software (IONDEV Srl, București, Romania) that was also used for the visualization of the data. The theoretical curves were created by QtiPlot FitWizard (IONDEV Srl, București, Romania) that produced mean values and errors as an output. These values, namely *k*_obs_ means and SD, were shown on the bar graphs in Figure 2B and Figure 5B.

## 4. Conclusions

The research on interactions of enzymes with various damaged DNA molecules by DEER spectroscopy and pre–steady-state kinetic analysis should make a considerable contribution to the understanding of the exact reasons for the substrate specificity of AP endonucleases and should clarify the mechanisms behind fine conformational rearrangements of the enzyme and substrate during their interaction. Previously reported theoretical data [31,32] suggest that a factor important for the substrate specificity of APE1-like AP endonucleases is conformational rigidity of the protein loop containing amino acid residues 253 to 257 of zAPE1, which correspond to residues 229–233 of hAPE1. To test this hypothesis, mutant zAPE1 enzymes containing substitution N253G, A254G, or E260A were obtained here. The properties of the WT enzyme and of the three mutants were analyzed by PAGE and stopped-flow approaches toward damaged DNA substrates, i.e., ODNs containing an F-site, DHU, dU, αA, or εA. The experiments showed that all the mutant enzymes retain AP endonuclease activity, although the activity of enzymes A254G and E260A is severalfold lower relative to the WT. The results showed that all the tested enzymes share a similar three-stage reaction mechanism. From the results of experiments with NIR substrates, it can be concluded that in comparison with the WT enzyme, mutant zAPE1 A254G possesses increased activity only toward DHU- and dU-substrates. By contrast, mutant E260A has much higher activity toward all the tested NIR substrates. Examination of conformational changes in DNA containing damaged bases clearly revealed multistep DNA rearrangements during the formation of an enzyme–substrate complex and its transformation into the catalytically active state. These structural rearrangements of DNA are directly associated with the capacity of damaged DNA for enzyme-induced bending and unwinding, which are required for eversion of the damaged nucleotide from the DNA duplex and its placement into the active site of the enzyme. Taken together, our findings experimentally proved the factors that control the substrate specificity of AP endonuclease zAPE1, and these results may be extrapolated to other APE1-like enzymes.

## Figures and Tables

**Table 1:** Dissociation constants of the enzyme–substrate complexes and observed rate constants characterizing the interaction of zAPE1 enzymes with the F-substrate ^a^.

Parameters	WT	N253G	A254G	E260A
*k*_1_, s^−1^	91	85	N/A	N/A
A_1_	−0.42	−0.39	N/A	N/A
*k*_2_, s^−1^	20	17.4	7.5	1.6
A_2_	1.63	1.6	1.2	1.0
*K*_a_, µM^−1^	3 ± 0.7	2.5 ± 0.5	2 ± 0.4	0.9 ± 0.2

N/A: not applicable owing to a negligible change of the signal. ^a^ Error of the rate constant did not exceed 10% for all constants unless otherwise stated.

**Table 2:** Dissociation and rate constants characterizing interaction of zAPE1 mutants with the F-substrate.

Constants	N253G	A254G	E260A
*k*_1_, μM^−1^s^−1^	29 ± 2	24 ± 3	11 ± 2
*k*_−1_, s^−1^	12 ± 2	12 ± 2	13 ± 3
*K*_a_ *, μM^−1^	2.5 ± 0.5	2.0 ± 0.4	0.9 ± 0.2
*k_cat_*, s^−1^	13 ± 2	8 ± 1	4.0 ± 0.5
*K_p_*, μM	4 ± 1	7 ± 1	10 ± 2

* *K*_a_ = *k*_1_/*k_−_*_1._

**Table 3:** Parameters of distance distribution functions (*r_max_*, standard deviation *δr*, and fraction *A*) obtained by three-Gaussian Monte Carlo simulation (see the Section 3.5).

Sample	*A*, %	*r_max_*, nm	*δr*, nm
Undamaged DNA ^a^	100	3.94 ± 0.02	0.36 ± 0.02
Undamaged DNA–zAPE1 complex	24 ± 2/72 ± 2	3.94 ± 0.02/4.60 ± 0.02	0.36 ± 0.02/0.34 ± 0.02
αA-substrate ^a^	100	4.37 ± 0.03	0.33 ± 0.03
αA-substrate–zAPE1 complex	100	4.50 ± 0.02	0.40 ± 0.02
F-substrate ^a^	65 ± 8/35 ± 8	3.45 ± 0.05/3.98 ± 0.06	0.32 ± 0.04/0.32 ± 0.04
F-substrate–zAPE1 complex	68 ± 7/31 ± 7	3.86 ± 0.04/4.51 ± 0.03	0.48 ± 0.05/0.26 ± 0.06

^a^ The data on duplexes without zAPE1 were taken from [45].

**Table 4:** Structures of the modified nucleotides and sequences of the DNA duplexes *.

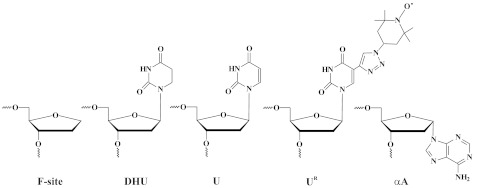	
Shorthand	Sequences of DNA Duplexes
X-substrate (FRET) X = C, F-site, DHU, dU, N = G X = αA, εA, N = T	5′-**FAM**-GCTCA**X**GTACAGAGCTG-3′ 3′-CGAGT**N**CATGTCTCGAC-**BHQ1**-5′
X-substrate (DEER) X = F-site, αA or A	5′-CG**U^R^**CTCTGTACATG**U^R^**GC-3′ 3′-GCAGAGAC**X**TGTACACG-5′

* FAM is 6-carboxyfluorescein, BHQ1 is black hole quencher, F is (2*R*,3*S*)-2-(hydroxymethyl)-3-hydroxytetrahydrofuran, αA is the α-anomer of 2′-deoxyadenosine, DHU is 5,6-dihydro-2′-deoxyuridine, and U^R^ is the TEMPO derivative of 2′-deoxyuridine.

## Data Availability

Experimental data are available upon request to N.A.K. Tel.: +7-383-363-5174, E-mail: ni-kita.kuznetsov@niboch.nsc.ru.

## Supplementary Materials

The supporting information can be downloaded at: https://www.mdpi.com/article/10.3390/ijms241411474/s1.
