# Supplementary material for: Inner Amino Acid Contacts Are Key Factors of Multistage Structural Rearrangements of DNA and Affect Substrate Specificity of Apurinic/Apyrimidinic Endonuclease APE1
Source: Int J Mol Sci. 2023 Jul 14;24(14):11474. doi: 10.3390/ijms241411474 (PMC10380840; doi:10.3390/ijms241411474)
Supplement: Supplementary file 1 [file ijms-24-11474-s001.zip › ijms-2501207-supplementary.pdf]

Supplementary materials

Inner amino acid contacts are key factors of multistage structural rearrangements of DNA and affect substrate specificity of apurinic/apyrimidinic endonuclease APE1

Anatoly A. Bulygin[1], Victoria N. Syryamina[2], Aleksandra A. Kuznetsova[1], Darya S. Novopashina[1], Sergei A. Dzuba[2], Nikita A. Kuznetsov[1,3]*

[1]Institute of Chemical Biology and Fundamental Medicine, SB RAS, Novosibirsk 630090, Russia;

[2] Institute of Chemical Kinetics and Combustion, SB RAS, Novosibirsk 630090, Russia;

[3]Department of Natural Sciences, Novosibirsk State University, Novosibirsk 630090, Russia

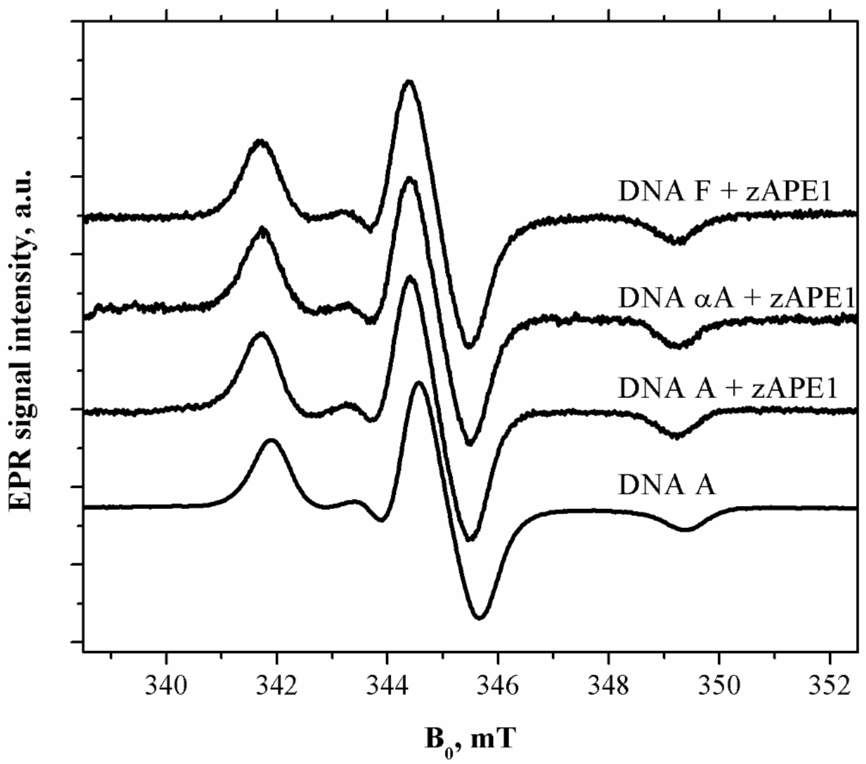

Figure S1. CW EPR spectra at 100 K for probed DNA duplexes. The data for DNA A are taren from ref. (1).

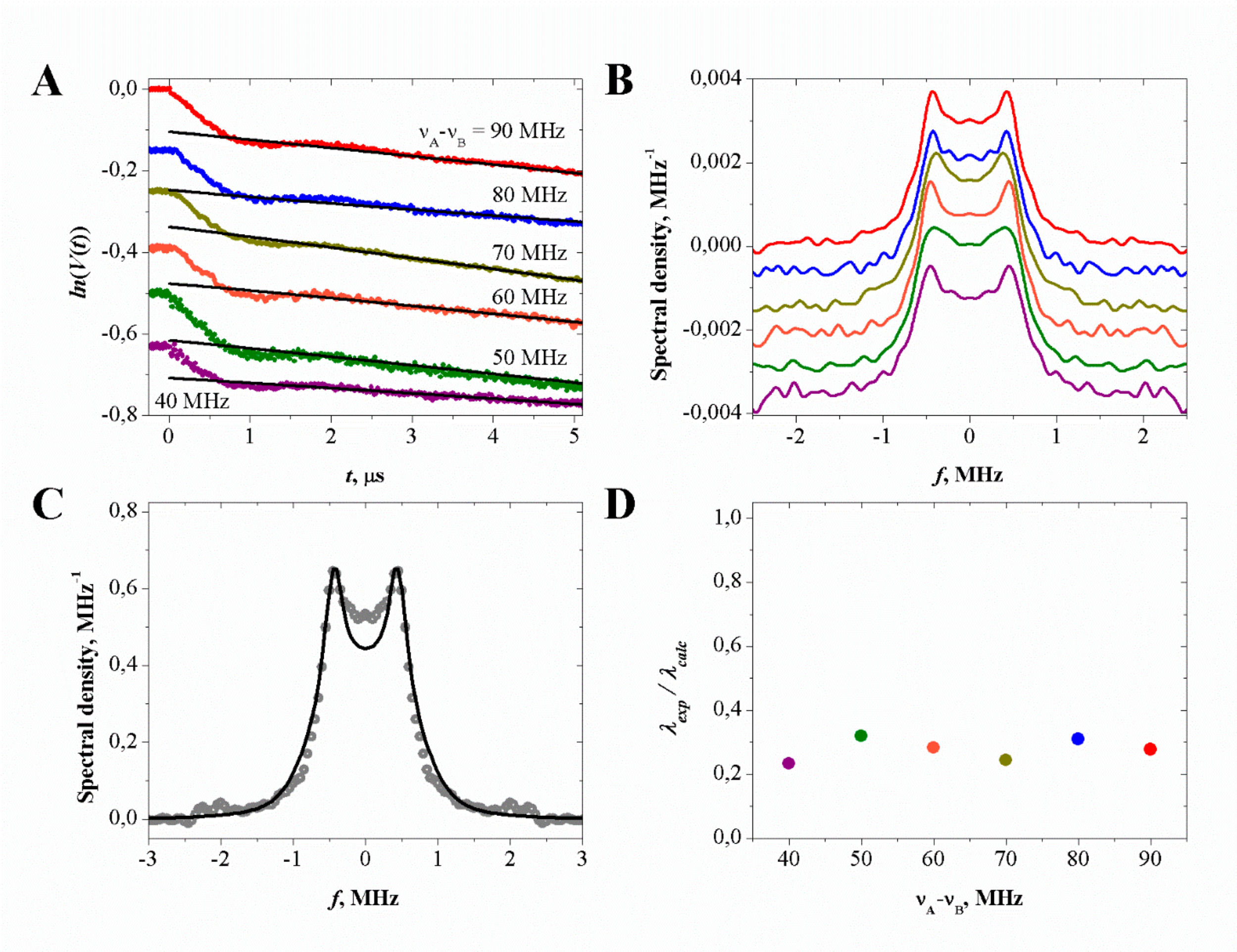

Figure S2. (A) Primary DEER traces for undamaged DNA-zAPE1 complex at different frequency offsets in the time domain. Decays are shifted down for clarity. Black lines shows the background decay. (B) Cosine-Fourier spectra for normalized DEER traces. The color code is same as in panel A). (C) The averaged experimental spectrum in frequency domain (circles) and its best fit (red curve). (D) The experimental *vs* calculated modulation depth λ at different frequency offsets. The color code the same as in panel A).

Expected excitation parameter λ_{calc} was calculated by convolution of the EPR spectrum with the excitation pulse profile as described elsewhere (2), and experimentally observed λ_{exp} excitation parameter was determined as value of the intramolecular decay $V_{inter}(t)$ at $t \rightarrow \infty$, which coincides with the value of background decay at t=0.

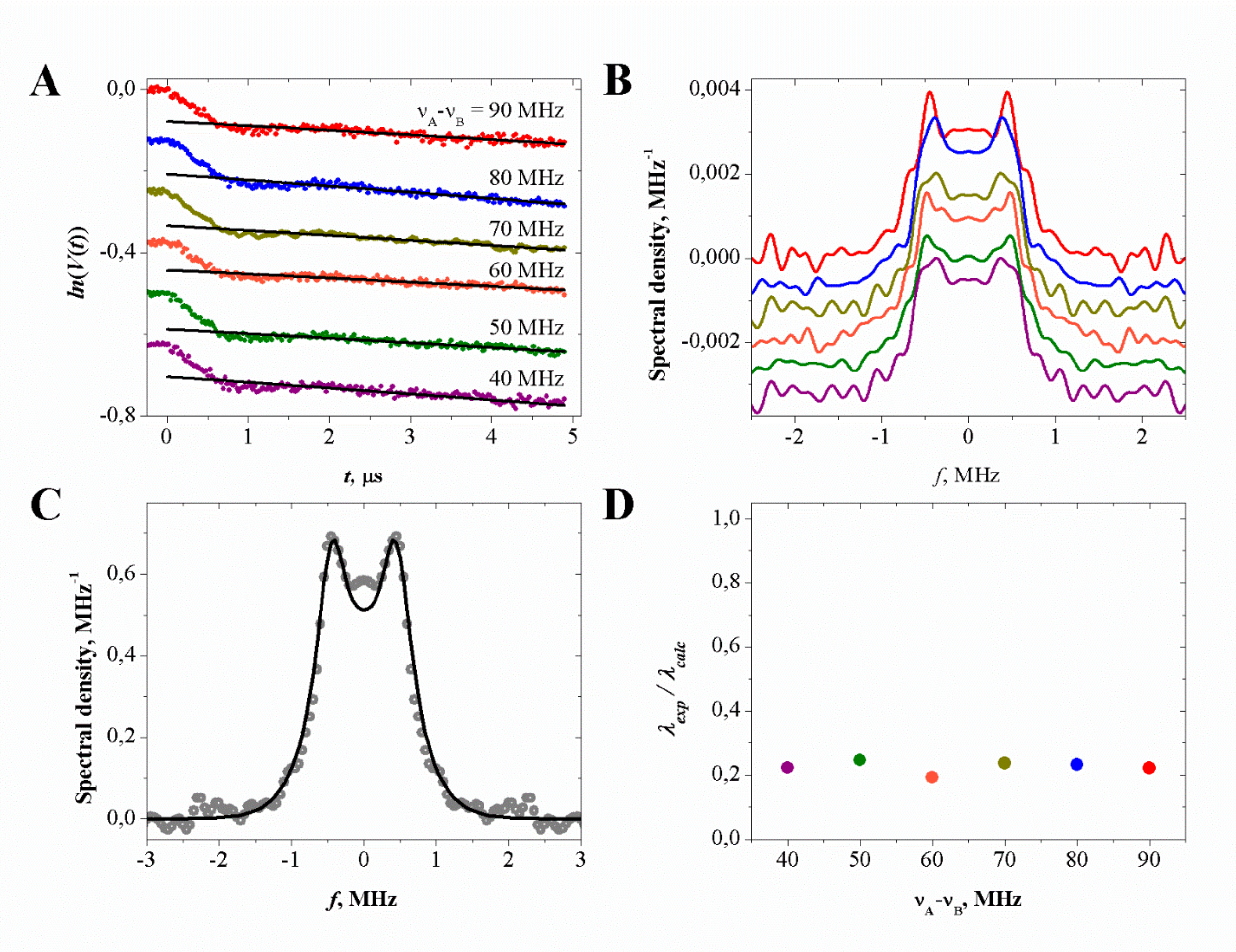

Figure S3. (A) Primary DEER traces for αA-substrate–zAPE1 complex at different frequency offsets in the time domain. Decays are shifted down for clarity. Black lines shows the background decay.

(B) Cosine-Fourier spectra for normalized DEER traces. The color code is same as in panel A).

(C) The averaged experimental spectrum in frequency domain (circles) and its best fit (red curve).

(D) The experimental *vs* calculated modulation depth at different frequency offsets. The color code the same as in panel A).

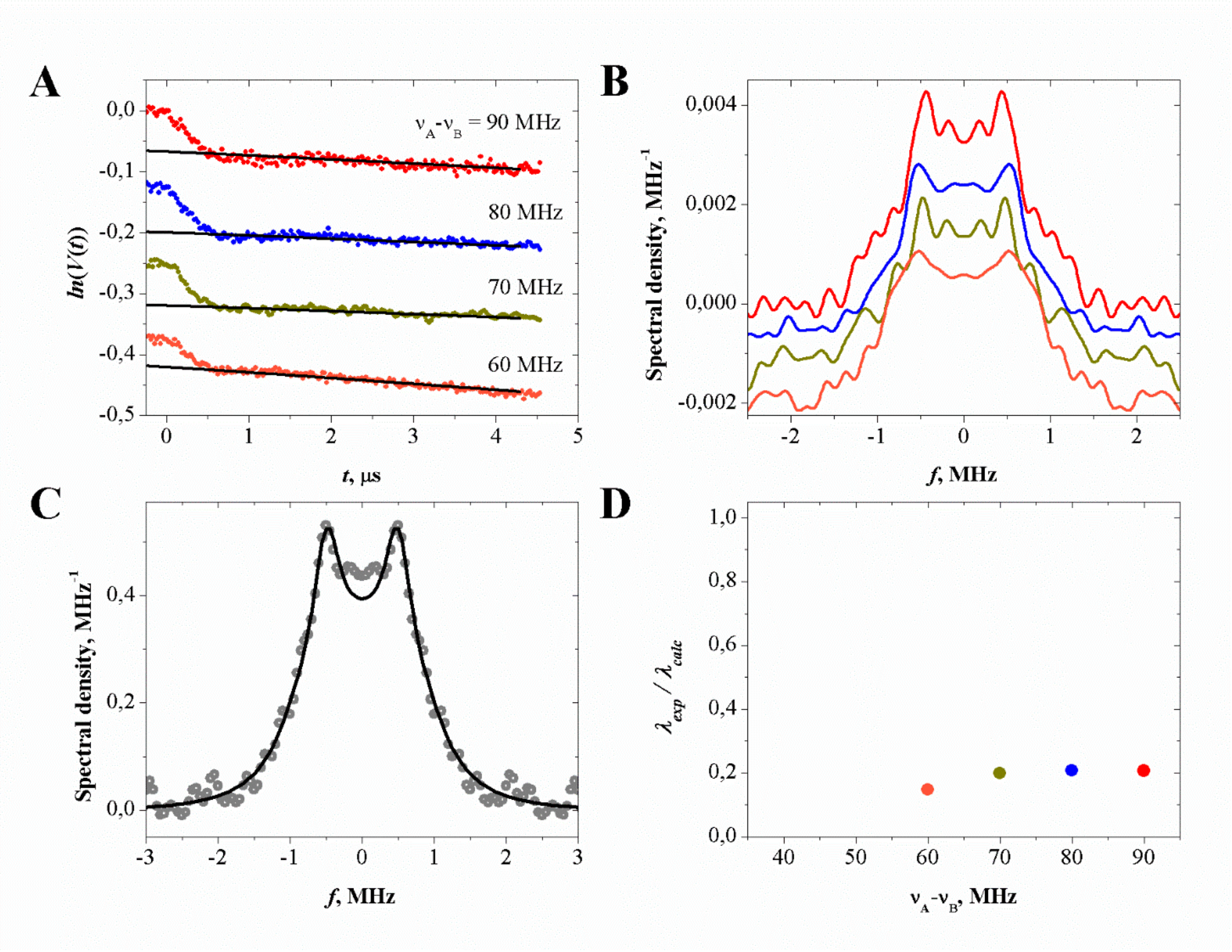

Figure S4. (A) Primary DEER traces for F-substrate-zAPE1 complex at different frequency offsets in the time domain. Decays are shifted down for clarity. Black lines shows the background decay.

(B) Cosine-Fourier spectra for normalized DEER traces. The color code is same as in panel A).

(C) The averaged experimental spectrum in frequency domain (circles) and its best fit (red curve).

(D) The experimental *vs* calculated modulation depth at different frequency offsets. The color code the same as in panel A).

References

1. Senchurova,S.I., Syryamina,V.N., Kuznetsova,A.A., Novopashina,D.S., Ishchenko,A.A., Saparbaev,M., Dzuba,S.A., Fedorova,O.S., Kuznetsov,N.A. (2022) The mechanism of damage recognition by apurinic/apyrimidinic endonuclease Nfo from Escherichia coli. *BBA - General Subjects*, doi: 10.1016/j.bbagen.2022.130216.

2. Tsvetkov,Y.D., Milov,A.D. and Maryasov,A.G. (2008) Pulsed electron–electron double resonance (PELDOR) as EPR spectroscopy in nanometre range. *Russ. Chem. Rev.*, **77**, 487–520.
